# Tibia bone properties at different time course of ovariectomized rats

**DOI:** 10.1186/s40200-014-0091-4

**Published:** 2014-09-02

**Authors:** Zairin Noor, Nia Kania, Bambang Setiawan

**Affiliations:** Research Center for Osteoporosis, Department of Orthopaedic and Traumatology, Ulin General Hospital, Medical Faculty, Lambung Mangkurat University, Jl. A. Yani Km 2 No.43, Banjarmasin, South Kalimantan Indonesia; Research Center for Osteoporosis, Department of Pathology, Ulin General Hospital, Medical Faculty, Lambung Mangkurat University, Banjarmasin, South Kalimantan Indonesia; Research Center for Osteoporosis, Department of Medical Chemistry and Biochemistry, Medicine Faculty, Lambung Mangkurat University, Banjarmasin, South Kalimantan Indonesia

**Keywords:** Trabecular, Osteoblast, Osteoclast, Turn over, Osteoporosis

## Abstract

**Background:**

The model of bilaterally ovariectomized rats mimics the accelerated bone loss observed in postmenopausal women due to estrogen deficiency. Although calcium is main mineral in bone, previous study in human showed there is hypermineralization and higher calcium level in hydroxyapatite crystal structure from osteoporosis patients. This study was aimed to investigate the effect of time course ovariectomized on tibia bone turn over markers, mineral elements, hydroxyapatite crystale, mesostructure, and histomorphometry.

**Methods:**

A total of 30 Wistar female rats were randomly assigned into three groups (n = 10 each): control group, ovariectomy group follow up for one month and two month. All animals procedures was according to Animal Ethics Guidelines and approval by ethic committee of the Medical Faculty, Lambung Mangkurat University which obtained prior the study. Expression of osteocalcin (OC) and C-telopeptyde collagen type I (CTX) was analyzed by ELISA method. Tibia bone mineral element was measured using X-Ray Fluorescence. Hydroxyapatite crystale structure was analyzed using X-Ray Diffracttion. Mesostructure was determined using Scanning Electron Microscope. Histomorphometry was analyzed using BoneJ software analyzer. ANOVA test was used to analyze the different level of serum bone turnover markers and bone mineral elements.

**Results:**

Serum OC and CTX were significantly decrease in one month and two month after ovariectomized groups compared to sham-operated group (P < 0.05). The levels Ca, P, Fe, Cu, Zn, Ni, Ca/P, and Cu/Zn were not significantly different in all groups (P > 0.05). The structure of hydroxyapatite crystal in one month and two month after ovariectomized groups were different compared with sham-operated control group. Mesostructure of tibia bone after one and two month ovariectomized procedure significantly different than that in sham-operated rats. The level of trabecular volume were lower significantly on OVX-1 and OVX-2 groups compared with sham group (P < 0.05). The trabecular thickness and spacing were increase significantly on OVX-1 and OVX-2 groups compared with sham group (P < 0.05). The trabecular number were significantly decrease OVX-1 and OVX-2 groups than that sham group (P < 0.05).

**Conclusion:**

We found that two month after ovariectomized decrease serum osteocalcin but not change bone mineral elements in rats. Also, we found the difference of lattice parameter of hydroxyapatite crystale structure and trabecular properties which determined bone mesostructure.

## Introduction

Osteoporosis is characterized by a reduction in bone mass and the micro-architectural deterioration of bone tissue, resulting in bone fragility and an increase in susceptibility to fracture [1]. Bone loss occurs with increasing age in females is directly linked to loss of ovarian function. The pathophysiology of this “ovary-related’ bone loss is very complicated and cannot be simply explained by either increased bone resorption or decreased bone formation [2]. Ovariectomized rats and dogs have been used extensively in osteoporosis models [3]. The model of bilaterally ovariectomized rats mimics the accelerated bone loss observed in postmenopausal women due to estrogen deficiency [4]. Ovariectomized animals suffer from accelerated bone turnover, showing stimulated osteoclastic bone resorption, and reactive osteoblastic bone formation with a net result of bone loss [5].

Atomic mineral is the smallest component of trabecular bone. Each atomic mineral was able to substitute another atomic mineral due its similarity in the atomic radius [6]. Ren et al., [7] showed that Zn substitution on hydroxyl apatite crystal was found to inhibit crystal growth due to its smaller atomic radius as compared with Ca. Ovariectomized rats showed significant gradual increase in serum calcium and phosphorus level [8]. Also, blood zinc and copper levels in ovariectomized rats were significantly increased compared to the sham control [3]. Change of atomic mineral composition would be modify the hydroxyapatite crystal structure. Finally, all process have involved would be determined the mesostructure of trabecular bone. Although calcium is main mineral in bone, previous study in human showed there is hypermineralization and higher calcium level in hydroxyapatite crystal structure from osteoporosis patients [9,10].

There is no study to evaluate the effect of time course ovariectomized on tibia bone properties. We hypothesize that there is difference of bone mineral elements, bone turn over markers, bone microstructure, and boe histomorphometry in time course of ovariectomized rats compared with control.

## Material and methods

### Animal and treatments

Thirty adult albino female Wistar rats, weighing 150–200 g were used in this study. The animals were acclimatized for one week to our laboratory conditions prior to experimental manipulation and were exposed to a 12-h light and 12-h dark cycle at room temperature of 24°C. They had free acces to standard laboratory chow and water *ad libitum*. The animals were randomly assigned into three groups (n = 10 each): control group, ovariectomy group follow up for one month (OVX-1), and two month (OVX-2). All animals procedures was according to Animal Ethics Guidelines and approval by ethic committee of the Medical Faculty, Lambung Mangkurat University which obtained prior the study.

### Surgical procedure

Under ketamine (50 mg/kg) and xylacine (8 mg/kg) anesthesia, thirty four OVX groups underwent bilateral ovariectomy by ventral incisions and eight were sham-operated (control) [11]. At the end of the experiment, animals in all groups were sacrificed. Serum and bone tissues were removed.

### Tissue preparation

At the end of the treatment, rats in all groups were anesthetized; their blood was drawn by cardiac puncture. Both tibia were collected, weighed, and latter rinsed with physiological saline. All samples were stored at glutaraldehyde until analyzed.

### Analysis of bone turn over markers

The serum bone formation markers osteocalcin was measured using Rat Osteocalcin/Bone Gla Protein OT/BGP ELISA kits from NovaTeinBio, Inc (Cambridge, MA, USA). The serum bone resorption marker C-telopeptide of type I collagen kit was purchased from NovaTeinBio, Inc (Cambridge, MA, USA).

### Analysis of bone mineral elements

Calcium (Ca), phosphorus (P), iron (Fe), copper (Cu), zinc (Zn), nickel (Ni) levels was evaluated by X-Ray Fluorescence (XRF). For XRF analysis, the tibia bones inserted in bone tube, then put in proper place in equipment. The processed bones were then analyzed at 20 kV accelerating voltage by a XRF (PANalytical MiniPAL 4) [12].

### Analysis of bone hydroxyapatite crystale

Characterization of the X-ray diffraction results was performed by means of PANanalytical X’Pert PRO-MPD, for ovariectomized and sham-operated rats proximal tibia. Subsequent analysis was by means of the software programs High Score Plus, Crystal Maker and DDVIEW, complemented with the latest version of PDF2. Diffraction spectra were recorded at an angle of 2θ, from 200 to 60o, with a Cu-K α radiation source (wave length = 1.54056 Å, 40 mA, 40 kV) and step size of 0.05° [13].

### Analysis of bone mesostructure

Mesostructure analysis was evaluated by Scanning Electron Microscope (SEM). For SEM evaluation, tibia from all groups were cut vertically from the proximal metaphysis area. Then the tibia bones were fixed with phosphate formalin buffer, dehydrated with graded concentration of ethanol and coated with gold and palladium. The processed bones were then analyzed at 20 kV accelerating voltage by a SEM (FEI Inspect TM S50) [12].

### Analysis of bone histomorphometry

Histomorphometric measurements were carried out according previous study with modification [13]. Measurements at distal tibia were made at 500x objective magnification using a scanning electron miscroscope (FEI Inspect TM S50) than analyzed by an image analyzer (BoneJ, USA). The parameters measured in this study were trabecular bone volume, trabecular thickness, trabecular number, and trabecular separation. Trabecular bone volume (BV/TV) is the number of trabecular bone within the spongy space (expressed as a percentage). Trabecular thickness (Tb.Th, in micrometers) was derived from trabecular perimeter (B.Pm) and B.Ar (Tb.Th = 1.99 B.Ar/2/B.Pm). Trabecular number (Tb.N, expressed per millimeter) and trabecular separation (Tb.Sp, expressed per micrometer) were calculated assuming that trabecular bone can be modeled by the parallel plates and bar model (Tb.N = Tb.Ar 10/Tb.Th; Tb.Sp = 1000/Tb.N − Tb.Th). All the formula, symbols, units, and nomenclature used for bone histomorphometry were in accordance to the guidelines by the American Society for Bone and Mineral Research (ASBMR) Histomorphometry Nomenclature Committee [14].

### Ethics

This research has been approved by research ethics committee Medical Faculty University of Lambung Mangkurat, Banjarmasin, South Kalimantan, Indonesia.

### Statistical analysis

Data are presented as mean ± SD and differences between groups were analyzed using ANOVA test using SPSS 16.0 statistical package. p < 0.05 was considered statistically significant.

## Results

### Bone turn over markers

Serum osteocalcin was significantly decrease in one month and two month after ovariectomized groups compared to sham-operated group (p < 0.05). Besides, serum C-telopeptyde collagen type I was significantly lower in one month and two month after ovariectomized groups compared to sham operated group (p < 0.05). All data shown in Table 1.

**Table 1:** **Levels of bone turnover markers in ovariectomized rats groups and sham-operated rats (ng/ml)**

**Level (ng/ml)**	**Sham**	**OVX-1**	**OVX-2**
CTX	1.457 ± 0.173	1.077 ± 0.206^a^	1.040 ± 0.066^a^
Osteocalcin	2.353 ± 0.122	1.790 ± 0.318^a^	1.631 ± 0.064^ab^

Values are presented as mean ± SD; OVX: ovariectomized rats; ^a^P < 0.05 in comparison with sham operated control group; ^b^P < 0.05 in comparison with one month ovariectomized rats group.

### Bone mineral elements

Ca, P, Fe, Cu, Zn, Ni, Ca/P, and Cu/Zn levels were not significantly different in one month and two month after ovariectomized groups compared to control group (p > 0.05) as given in Table 2.

**Table 2:** **Levels of bone mineral elements in ovariectomized rats groups and sham-operated rats (%)**

**Level (%)**	**Sham**	**OVX-1**	**OVX-2**
Calcium	83.08 ± 7.17	82.82 ± 5.77	85.17 ± 2.34
Phosphorus	9.66 ± 3.32	8.33 ± 3.08	8.68 ± 2.90
Iron	1.17 ± 1.02	1.77 ± 0.61	0.92 ± 0.38
Copper	0.42 ± 0.45	0.41 ± 0.29	0.15 ± 0.03
Zinc	0.78 ± 0.28	0.97 ± 0.24	0.78 ± 0.17
Nickel	2.14 ± 4.21	2.88 ± 3.42	0.49 ± 0.74
Ca/P	9.96 ± 4.73	11.45 ± 5.63	12.03 ± 2.96
Cu/Zn	0.45 ± 0.31	0.42 ± 0.31	0.18 ± 0.01

Values are presented as mean ± SD; OVX: ovariectomized rats.

### Bone hydroxyapatite crystale

**Figure 1:**
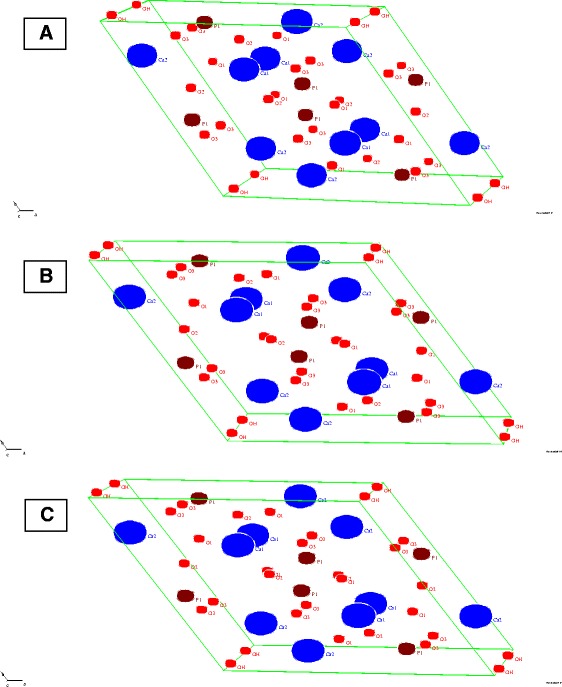
**Hydroxyapatite crystale of sham-operated rats (A) and one (B) and two month (C) after ovariectomized procedure.**

The atomic mineral composition in bone hydroxyapatite crystale structure in one month and two month after ovariectomized groups was not different compared with sham-operated control group. The structure of hydroxyapatite crystal in one month (Figure 1B) and two month (Figure 1C) after ovariectomized groups was different compared with sham-operated control group (Figure 1A). The lattice parameter of sham-operated rats (P 6_3/m; a = 9.4464; b = 9.4464; c = 6.8908) is different compared with one month (P 6_3/m; a = 9.4504; b = 9.4504; c = 6.9087) and two months (P 6_3/m; a = 9.4278; b = 9.4278; c = 6.8892) after ovariectomized.

### Bone mesostructure

**Figure 2:**
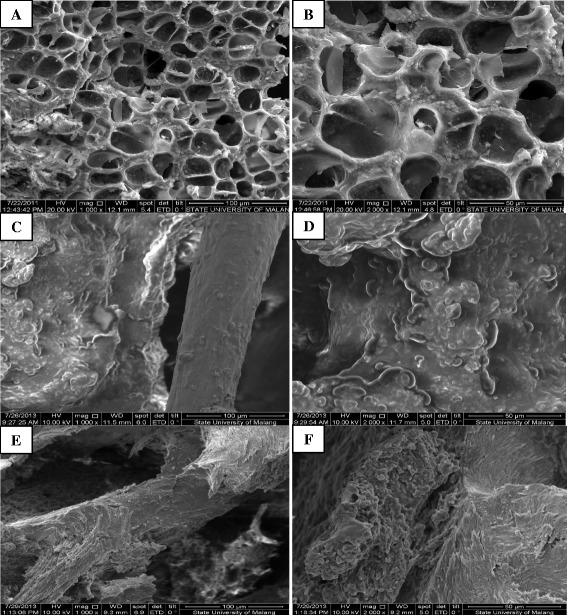
**Mesostructure of sham-operated rats (A) and one (B) and two month (C) after ovariectomized procedure.** Mesostructure of sham-operated rats presented rod like trabecules with honey comb appearance and minimal holes **(A and B)**. Mesostructure of tibia bone after one and two month ovariectomized procedure significantly different compared with sham-operated rats. At one month after ovariectomized procedure we found reduction of trabecular integrity, lacunae, and decreased thickening of trabecular **(C)**. The surface of trabecular showed granules structured **(D)**. We can see trabecular breaking and stump structure, which contributed to massive hole, was found in tibia bone at two month from ovariectomized procedure **(E)**. Beside, the loosing of granule structure are observed **(F)**.

Mesostructure of sham-operated rats presented rod like trabecules with honey comb appearance and minimal holes as shown in Figure 2A and B. Mesostructure of tibia bone after one and two month ovariectomized procedure significantly different compared with sham-operated rats. At one month after ovariectomized procedure we found reduction of trabecular integrity, lacunae, and decreased thickening of trabecular wall as seen in Figure 2C. The surface of trabecular showed granules structured to see in Figure 2D. In Figure 2E, we can see trabecular breaking and stump structure, which contributed to massive hole, was found in tibia bone at two month from ovariectomized procedure. Beside, the loosing of granule structure also observed (Figure 2F).

### Bone histomorphometry

Table 3 present the level of trabecular volume, thickness, spacing, and number in sham, OVX-1, and OVX-2 group, respectively. The level of trabecular volume were lower significantly on OVX-1 and OVX-2 groups compared with sham group (P < 0.05). The trabecular thickness and spacing were increase significantly on OVX-1 and OVX-2 groups compared with sham group (P < 0.05). The trabecular number were significantly decrease OVX-1 and OVX-2 groups than that sham group (P < 0.05).

**Table 3:** **Bone histomorphometry in ovariectomized rats groups and sham-operated rats**

**Level**	**Sham**	**OVX-1**	**OVX-2**
BV/TV (%)	0.674 ± 0.001	0.411 ± 0.004^a^	0.646 ± 0.001^ab^
Tb.Th (μm)	1.479 ± 0.001	2.789 ± 0.319^a^	2.072 ± 0.010^ab^
Tb.Sp (μm)	0.377 ± 0.003	1.364 ± 0.002^a^	0.525 ± 0.000^ab^
Tb.N (mm^−1^)	538.535 ± 0.475	234.391 ± 0.165^a^	385.863 ± 1.478^ab^

Values are presented as mean ± SD; OVX: ovariectomized rats; ^a^P < 0.05 in comparison with sham operated control group; ^b^P < 0.05 in comparison with one month ovariectomized rats group.

## Discussion

Bone matrix can be considered a composite material, comprised of mineral and organic phases. The mineral phase largely accounts for the stiffness of bone [15], whereas the organic phase, mainly constituted of type I collagen, provides bone its ductility and toughness, i.e. its ability to undergo deformation and absorb energy after it begins to yield [16,17]. Bone collagen can also undergo a series of nonenzymatic transformations including the advanced glycation end products and the isomerization of aspartic acid residues within the C-telopeptides, which have also been shown to be associated with bone mechanical properties in ex-vivo experiments [18–22]. In this study the level of CTX decrease significantly in one and two months after ovariectomized compared to sham control, but not difference between ovariectomized groups. This reduction indicated that cross linked to collagen as dominant organic molecules in bone is decreased or indicated rupture of crosslinks in the collagen fibrils [23].

Osteocalcin was a protein secreted by osteoblast as indicator of osteoblast activity. In this study, we found a decreasing of osteocalcin as indicator of reduction of osteoblast activity to secrete protein due to low population or low activity of osteoblast. These decreased levels of serum osteocalcin, are consistent with histological studies reporting decreased osteoblast number [24]. After secretion of newly synthesized intact osteocalcin by osteoblastic cells, part of the molecule is secreted in the blood and part is incorporated in bone tissue, through the binding of its gamma carboxyglutamic acids to hydroxyapatite. Thus, if the fraction of newly synthesized osteocalcin captured into bone is altered – because of lattice parameter in the hydroxyapatite crystal structure – this would also influence circulating levels. In this study we found no difference of mineral elements and mineral composition in hydroxypatite crystale structure, but the lattice parameter is changed. This finding indicated that ovariectomized modify the arrangement of atomic mineral in in hydroxypatite crystale. Subsequently this mineral phase transformations or rearrangements modification [25] would determined bone mesostructure.

This study showed that Ca, P, Fe, Cu, Zn, Ni, Ca/P, and Cu/Zn levels in ovariectomized rats was not change significantly different compared to control rats. Previous studies showed that bone calcium and total mineral element will decreased after one month ovariectomized [25,26]. In this study, we found contradictive with literature. Our finding indicated that mineralization is adaptive homeostatic process to compensate the effect of estrogen deficiency. Although reduced in mass, the bones are normal with respect to mineralization. In addition, substitution of atomic mineral may also contribute to bone mineralization [27].

Mesostructure of sham-operated rats presented rod like trabecules with honey comb appearance and minimal holes. At one month after ovariectomized procedure we found reduction of trabecular integrity, lacunae, and decreased thickening of trabecular wall. The surface of trabecular showed granules structured. The trabecular breaking and stump structure, which contributed to massive hole, was found in tibia bone at two month from ovariectomized procedure. Beside, the loosing of granule structure also obeserved. We also found that trabecular volume were lower significantly on OVX-1 and OVX-2 groups compared with sham group (P < 0.05). This finding indicated that ovariectomized increased the porocity of trabecular. The changes of lattice parameter in hydroxyapatite crystale determined the porocity of trabecular bone. In this study, the trabecular thickness and spacing were increase significantly on OVX-1 and OVX-2 groups compared with sham group (P < 0.05). Besides, the trabecular number were significantly decrease OVX-1 and OVX-2 groups than that sham group (P < 0.05). This finding partially confirmed previous study reported that ovariectomized rats exhibit significant decreases in the thickness of the cortex and the number and size of trabecular [8]. Resorption process was also observed to be intensified in the examined trabecular bone [28].

## Conclusions

We found that two month after ovariectomized decrease serum osteocalcin but not change bone mineral elements in rats. Also, we found the difference of lattice parameter of hydroxyapatite crystale structure and trabecular properties which determined bone mesostructure.
